# Covered Stent Fenestration for Side Branch Salvage in Bifurcation Coronary Perforation: A Systematic Bench-Test Evaluation

**DOI:** 10.1016/j.jscai.2026.105521

**Published:** 2026-07-28

**Authors:** Troels Yndigegn, Per Bondesson, Göran K. Olivecrona, Henrik Wagner, Sasha Koul, Matthias Götberg, Helle Soeholm, David Erlinge, Mihai Cocoi, Olivier Gach, Adrien Jossart, Quentin Trefois, Agostino Spano, Giuseppe Colletti, Gregor Leibundgut, Marouane Boukhris, Claudiu Ungureanu, Moman A. Mohammad

**Affiliations:** a Division of Cardiology, Department of Clinical Sciences Lund, Lund University, Lund, Sweden; b Department of Cardiology, Skåne University Hospital, Lund, Sweden; c Department of Cardiology, “Niculae Stancioiu” Heart Institute, Cluj-Napoca, Romania; d Department of Cardiology, MontLégia, Liege, Belgium; e Department of Cardiology, CHU Helora, Jolimont Hospital, La Louviére, Belgium; f Department of Cardiology, Clinique Saint Joseph, Vivalia, Arlon, Belgium; g Department of Cardiology, University Hospital Basel, Basel, Switzerland; h Department of Cardiology, CHU Saint-Etienne, Saint-Etienne, France

**Keywords:** complex percutaneous coronary intervention, complications, covered stent, perforation

## Abstract

**Background:**

Coronary perforation at a bifurcation level is a life-threatening emergency. Covered stents may seal the defect but risk side-branch occlusion. We evaluated the feasibility and technical considerations of guide wire fenestration at coronary bifurcations and present a case where a covered side branch was successfully salvaged.

**Methods:**

A transparent bifurcation phantom replicating coronary anatomy was constructed and integrated into a pulsatile flow system. Three fenestration strategies were evaluated: (1) a balloon-blocking approach, in which inflation of a balloon in the main branch after covered stent deployment was hypothesized to facilitate wire redirection and fenestration; (2) a microcatheter-assisted approach; and (3) a wire-only approach. These strategies were tested by 15 percutaneous coronary intervention operators with varying levels of experience. Seven guide wires with differing characteristics and tip loads ranging from 3 to 12 g were used.

**Results:**

The balloon occlusion strategy achieved fenestration in all tests (n = 15) using both 12 g and 3 g wires, as did microcatheter-assisted fenestrations (n = 2). Wire-only strategy succeeded in 14 of 17 attempts across wires ranging from 3 to 12 g; however, 3 of 6 attempts using 3 g wires required conversion to balloon occlusion. Median time to fenestration was 69 seconds for both balloon occlusion and microcatheter strategies, compared with 81 seconds, 94 seconds, and 167 seconds for wire-only approaches using 12 g, 6 g, and 3 g wires, respectively. Angiography and micro–computed tomography demonstrated satisfactory results, but endoscopic analysis revealed polyurethane membrane recoil reducing the ostial area by up to 75%, necessitating side-branch stenting. All evaluated side-branch stenting techniques improved the ostial lumen area.

**Conclusions:**

Covered stent fenestration is a feasible bailout strategy for preserving side-branch patency in critical bifurcation perforations.

## Introduction

The treatment of complex coronary lesions, particularly those involving the left main (LM) stem, has become increasingly common and technically demanding in contemporary interventional cardiology.^1^, ^2^, ^3^ This trend is driven by the expanded use of percutaneous coronary intervention (PCI) in older and frailer patients who are more susceptible to complications, as well as by a greater burden of calcified coronary disease, often necessitating specialized plaque-modifying techniques such as rotational or orbital atherectomy or intravascular lithotripsy.^4^ Although lesion preparation enhances stent deployment and outcome, it also increases the risk of coronary artery perforation.^5^ In the context of LM bifurcations, the consequences of such a complication are especially grave. Unlike perforations in straight or distal coronary segments, where a covered stent can typically seal the defect without compromising major flow, a perforation at the LM bifurcation poses a unique challenge: covered stent implantation, although potentially life-saving, may result in immediate occlusion of a major side branch (SB) such as the left anterior descending (LAD) or left circumflex (LCx) artery, leading to catastrophic outcomes. In such high-stakes scenarios, there is a critical need for the development of a rapid technique that enables restoration of SB patency immediately after covered stent deployment. We, therefore, designed a dedicated bench model that accurately replicates a coronary bifurcation, allowing for controlled and detailed evaluation of : (1) a simple and novel balloon-blocking strategy, (2) a microcatheter strategy, and (3) a guide wire-only strategy to fenestrate a covered stent and to reestablish blood flow to the occluded SB rapidly.

## Methods

### In vitro model and prestudy assessment

A silicone-based bifurcation phantom was developed to replicate the anatomic and mechanical properties of human coronary arteries. The elastomer material closely mimics vascular compliance and elasticity, and its transparent structure permits real-time visualization of device behavior. The model incorporates a main branch (representing the LM) with a proximal inner diameter of 4.5 mm, tapering to 4.0 mm at the carina and 3.0 mm distally, and an SB of 3.5 mm tapering to 3.0 mm (representing the LAD and LCx). During pilot testing, direct wire advancement against the covered stent was ineffective. Successful fenestration required precise tip engagement against the stent strut or polyurethane membrane, identified by a subtle guide wire deflection followed by controlled rotational movement with gentle forward pressure, allowing the wire to drill through the membrane. Prestudy assessments confirmed that soft-tipped wires were insufficient and that a minimum tip load of approximately 3 g was required for reliable fenestration.

### Operator experience

A total of 15 operators with varying levels of interventional cardiology experience were included. Four operators were classified as high-volume experts, each performing more than 500 PCI annually, including complex chronic total occlusion procedures. Five operators were experienced interventionalists with over 10 years of practice and an annual volume of 200 to 300 PCI. The remaining operators were fellows, each having performed fewer than 300 PCI during their entire training period.

### Stents

The stent used in this study for sealing the main vessel was the PK Papyrus covered stent (Biotronik Inc), a cobalt-chromium prosthesis specifically engineered for the management of coronary perforations. It has a strut thickness of 60 μm for stent diameters <3.5 mm and 80 μm for diameters ≥3.5 mm. The stent metal frame is encapsulated in a single-layer, nonwoven biostable polyurethane polymer, designed to rapidly seal vessel breaches while maintaining high flexibility and trackability in tortuous anatomy. For this study, we utilized stents with a diameter ≥3.5 mm, deployed at a fixed pressure of 20 atm for all bench-test iterations. Each fenestration was done on a newly implanted stent. For the SB, we used conventional drug-eluting stents from different brands, measuring 3.0 mm in diameter and deployed at 18 atm.

### Study protocol

A total of 15 balloon-blocking strategy experiments were conducted. Two guide wires were selected when evaluating the balloon-blocking technique. These were the MIRACLEbros 3 and the Confianza Pro 12, both from Asahi Intecc (Japan). The Confianza 12 g guide wire was used in 10 tests, and the Miracle 3 g was used in 5. These wires were selected to represent a range of axial stiffness required for mechanical perforation of the polyurethane membrane of the covered stent to see if added stiffness increased the success of the experiment. In all tests, the distal tip of each guide wire was manually shaped by the operator approximately 2 mm from the end, with a mean angle of 60°. Operators were instructed to spin/drill the wire through the polyurethane membrane rather than attempt direct puncture by advancing the wire back and forth.

The procedure protocol of the balloon-blocking technique consisted of 3 major steps:

1. The bifurcation segment was positioned at the level of the angiographic x-ray emitter and flat-panel detector, after which guide wires were advanced in both branches (Figure 1A). The covered stent was then deployed in the main vessel (Figure 1B). Following stent deployment, the stent balloon was advanced so that the second radiopaque marker of the stent balloon aligned precisely with the ostium of the SB, at the level of the carina (Figure 1B, C).

**Figure 1:**
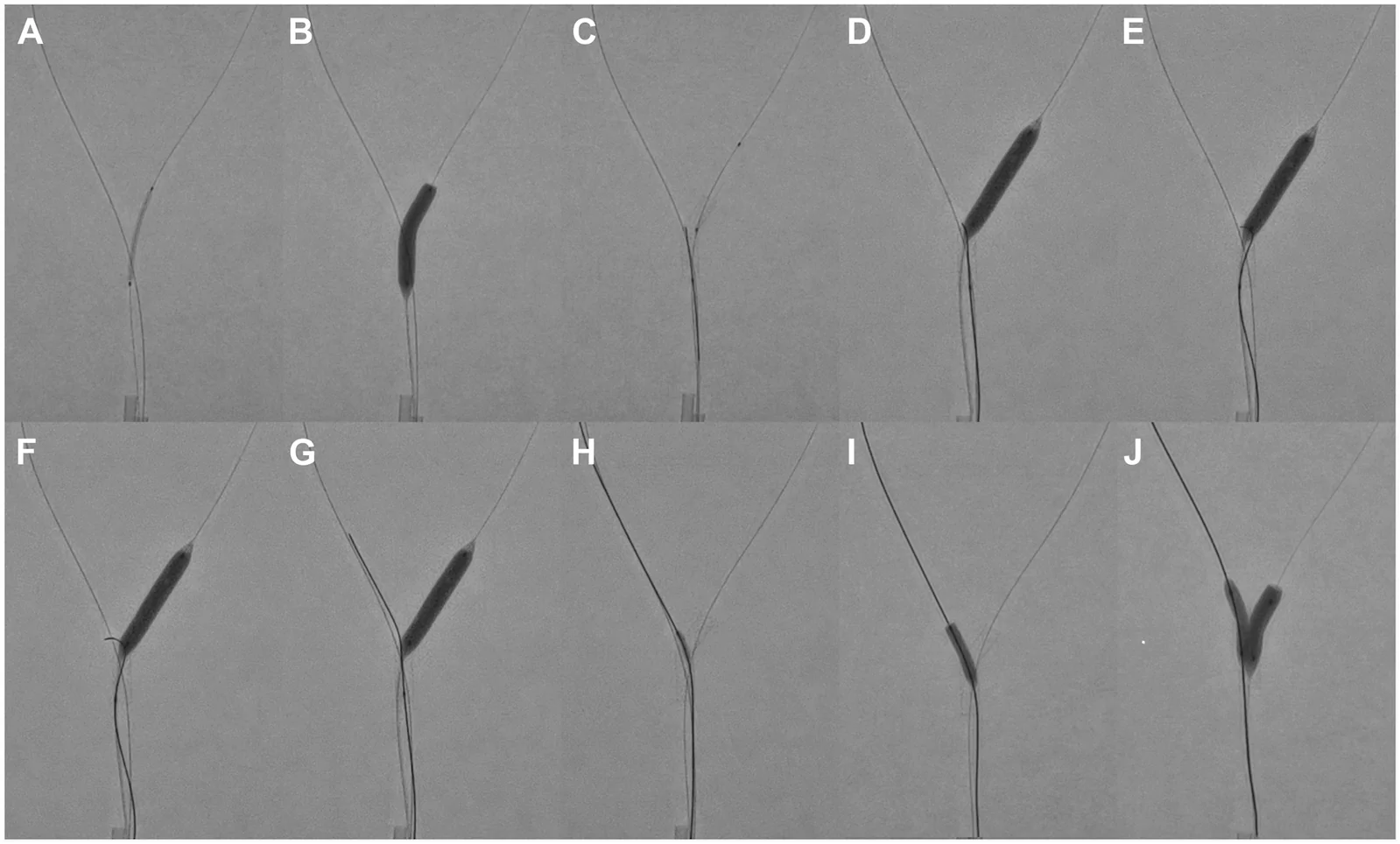
**Procedure steps of the balloon occlusion technique.** (**A**) and (**B**) Two guide wires are advanced, one into each branch of the bifurcation model, followed by deployment of the covered stent in the main vessel. (**C**) After stent deployment, the stent balloon is positioned so that its second radiopaque marker aligns with the side-branch ostium at the carina. A stiff guide wire with a low-profile balloon (1.2-2.0 mm) mounted proximally is advanced to the bifurcation. (**D**) Inflation of the covered stent balloon prevents distal passage of the guide wire during attempted puncture. (**E-G**) The guide wire tip engages the stent struts, and with a spinning motion and gentle forward pressure, fenestration of the stent membrane is achieved. (**H**) The support balloon is advanced and inflated. (**I**) A 1:1-sized noncompliant balloon is inflated. (**J**) Kissing balloon inflation is performed before proceeding to the placement of the stent in the side branch.
2. Covered stent wire fenestration: A stiff guide wire was advanced to the bifurcation level with a secondary low-profile balloon (1.2-2.0 mm) mounted on the guide wire to add support/increase the tip load of the guide wire and to fasten the procedure once the fenestration was performed. The covered stent balloon was then inflated, blocking the passage of the guide wire toward the distal main-vessel once puncture was attempted (Figure 1D, E).
3. SB recovery: Once successful guide wire fenestration of the covered stent was achieved (Figure 1E-G), the main objective was to obtain a large opening of the SB. Therefore, the procedure continued with the angioplasty of the SB ostium. After entering the SB with the guide wire, the occlusive balloon was deflated, and the low-profile balloon on the stiff guide wire was advanced through the covered stent and inflated (Figure 1H, I) and kissing ballon inflation was done (Figure 1J).

In addition to the balloon-blocking protocol, a total of 17 experiments with 2 different protocols were evaluated. A microcatheter strategy using the ReCross dual-lumen microcatheter (IMDS) with the Miracle 3 g guide wire was tested in 2 experiments, and guide wire fenestration without microcatheter or balloon-blocking technique using various wires with different tip loads was attempted in 15 experiments. For wire-only fenestration, a total of 7 high-penetration guide wires were evaluated. These were Halberd (Asahi Corp), Confianza Pro 12, MIRACLEbros 12, Judo 6, MIRACLEbros 6, MIRACLEbros 3, and Ultimate 3. Halberd and MIRACLEbros are high-penetration wires with different specifications. Although Halberd has an uncoated distal tip, MIRACLEbros 12 and 6 have a hydrophobic coating designed to drill through lesions. Judo 6 (Boston Scientific) is a high-penetration wire with a small tip of just 0.20 mm and a long hydrophilic taper aimed at facilitating lesion entry. The ULTIMATEbros 3 (Asahi Corp) is a hydrophilic wire with an uncoated distal tip. Finally, we assessed a jailed-balloon strategy, in which a balloon was overstented with the covered stent and subsequently inflated to create a microchannel permitting SB perfusion.

### Imaging tools and visualization modalities

All experiments were conducted in a clinical-grade catheterization laboratory equipped with a ceiling-mounted fluoroscopy system, providing high-resolution, real-time x-ray imaging for all procedural steps. Fluoroscopic guidance was used systematically during wire navigation, stent deployment, and balloon positioning, simulating authentic clinical conditions.

For detailed morphological assessment of the results, micro–computed tomography was used together with a digital camera with a macroobjective to inspect the stent strut deformation. A flexible 2 mm endoscope was used to capture intraluminal views from both the main-vessel and SB perspectives.

## Results

A total of 34 standardized bench tests were conducted: 15 using the balloon-blocking technique, 2 using a microcatheter-assisted approach, and 17 employing a wire-only strategy. Guide wire fenestration of the covered stent was followed by SB balloon crossing and inflation at the fenestration site, which was achieved in all 15 cases evaluating the balloon-blocking technique, regardless of the operator’s level of experience or wire tip load. The mean time from covered stent deployment to first SB balloon inflation was 236 seconds, with a median of 220 seconds for procedures using the blocking balloon strategy. The mean time for guide wire penetration through the covered stent membrane following main branch occlusion with balloon inflation was 69 seconds, with a median of 58 seconds. Specifically, the Confianza Pro 12 required a mean time of 70 seconds, with a median of 57 seconds, while the MIRACLEbros 3 g demonstrated a similar efficacy, with a mean penetration time of 67 seconds and a median of 62 seconds. The interval between achieved wire fenestration and subsequent SB balloon inflation was 47 seconds, with a median of 45 seconds. In 5 cases, repositioning of the occlusive balloon was necessary to optimize alignment; this occurred in 3 procedures performed by fellows and 2 by high-volume operators.

Two attempts using the ReCross microcatheter were achieved with a mean fenestration time of 69 seconds and a median of 52 seconds. The mean time from stent deployment to SB balloon inflation in this subgroup was 206 seconds, with a median of 216 seconds. This total lap time considered the ReCross microcatheter extraction, which needs a trapping balloon technique to avoid the loss of the guide wire distal position.

Seventeen wire-only fenestration attempts were performed, starting with wires with 12 g tip load and progressing in descending tip load if fenestration was achieved. Three fenestrations with Confianza Pro 12 were followed by 3 fenestrations with Halberd, and 3 with MIRACLEbros 12. All fenestrations with wires with a 12 g tip load were concluded. Fenestrations were achieved with both Judo 6 and MIRACLEbros 6, and 3 fenestrations were concluded with MIRACLEbros 3, and 3 unsuccessful attempts were made with ULTIMATEbros 3, which were converted to the balloon occlusion strategy, which was successful. Recorded times to fenestration were, on average, 167 seconds with MIRACLEbros 3 and 94 seconds for the wires with 6 g tip load, and 81 seconds for 12 g wires. Finally, we evaluated the jailed-balloon technique, which was able to produce a microchannel maintaining SB flow, after which wire-only fenestration could be accomplished.

### Evaluation of results

After fenestration of the covered stent, we proceeded with balloon dilatation, first using a low-profile 1.5 to 2.0 mm balloon followed by a noncompliant (NC) 1:1 sized balloon using multiple inflations. Fluoroscopy demonstrated overall satisfactory results. However, angiography revealed contrast passage through the fenestrated SB, albeit with reduced flow. Postprocedural endoscopic imaging offered additional insights into the morphological modifications of the PK Papyrus polyurethane membrane at the fenestration site. From the main-vessel lumen, stent struts appeared appropriately expanded, and the SB orifice appeared discrete with a geometrically defined shape. However, retrograde endoscopic visualization from the SB revealed substantial residual stenosis with the fenestrated membrane exhibiting significant recoil and collapse (Figure 2). This behavior, attributed to the elastic memory of the polyurethane, caused the membrane to partially drape over the orifice, creating a “curtain-drop” effect. No membrane flaps, floating fragments, or embolic events were observed in any of our experiments. The resultant recoil markedly reduced the effective cross-sectional area of the SB opening and produced visual stenosis, despite technically successful stent strut expansion. Quantitative image segmentation analysis showed that membrane recoil occluded up to 75% of the SB orifice area. Multiple cutting balloon inflations with a 1:1-sized cutting balloon were then tested without improvement in orifice area. To overcome this limitation, we postulated that a regular stent could withstand this recoil, and several modified 2-stent PCI techniques were employed, including adapted versions of culotte, TAP, and Internal Crush (Figure 2, Figure 3, Figure 4, Figure 5). Given that the main vessel had already been stented, these adaptations are referred to as PK-Culotte, PK-TAP, and PK-Crush, respectively. Quantitative image segmentation analysis after 2-stent PCI showed that there was significant recoil strangling the stent in the SB. The relative open orifice area was 54.5% with PK-Culotte, 46.4% with PK-TAP, and 40.1% with PK-Crush (Figure 6). Finally, we performed a 2-covered-stent bifurcation PCI for situations in which the perforation is at the bifurcation carina (Figure 7). This procedure required 2 sequential fenestrations: first from the main vessel into the SB, and subsequently, after side-branch stent implantation, from the SB back into the main vessel. Imaging analysis demonstrated acceptable patency in the SB; however, significant residual stenosis remained at the main-vessel ostium despite 1:1 balloon dilation. To address this, a standard drug-eluting stent (third) was implanted in the main vessel to mitigate recoil from the covered stent (Figure 7).

**Figure 2:**
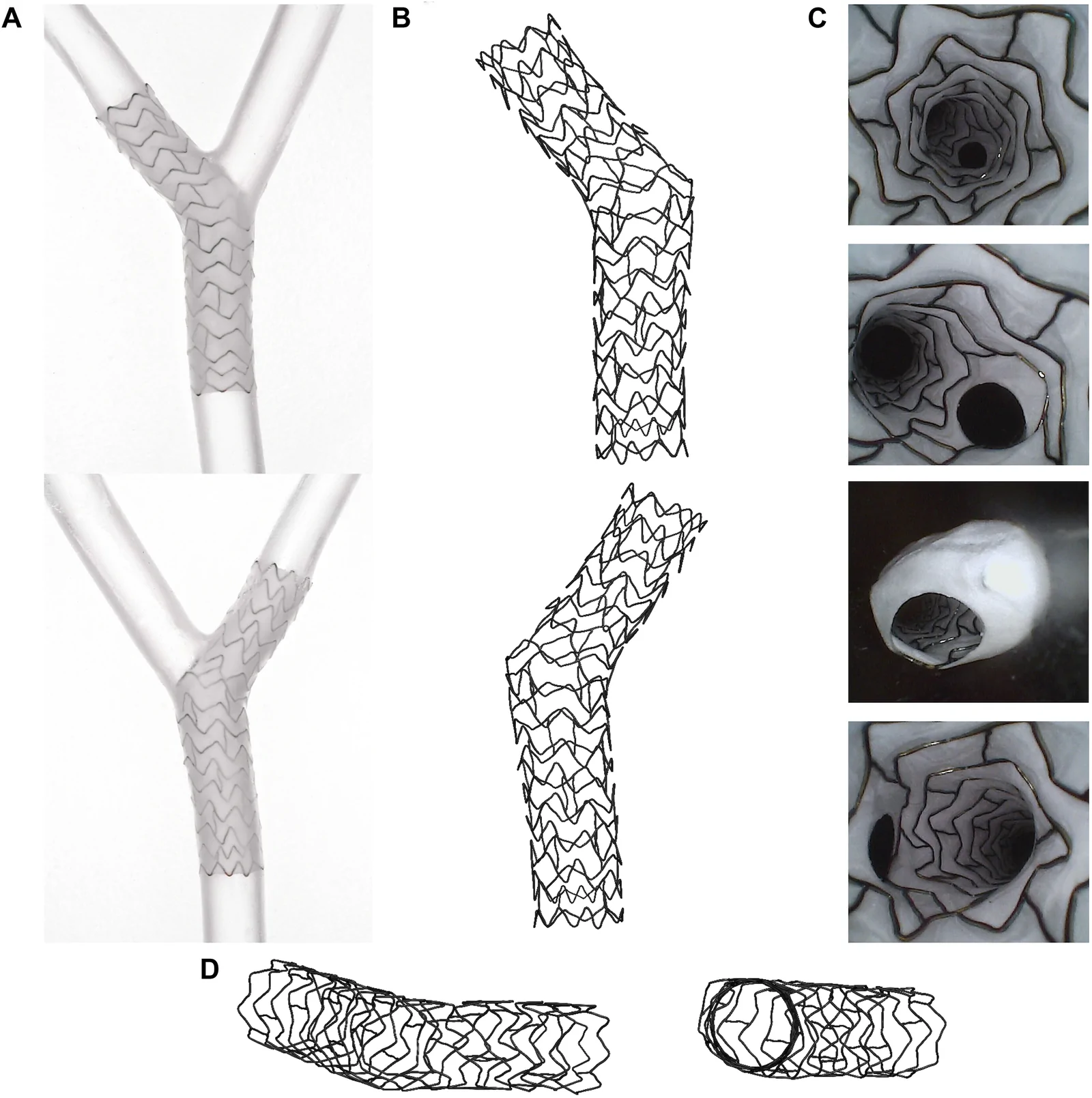
**Visual assessment of PK-provisional.** (**A**) Macroscopic images of the covered stent. (**B**) Micro–computed tomography images of the covered stent. Combined visual inspection and micro–computed tomography imaging demonstrated generally acceptable structural integrity. (**C**) Endoscopic evaluation. The upper 2 images depict views from the proximal main branch; the third image shows a retrograde view from the side branch; and the lowermost image presents a retrograde view from the main branch. Endoscopy revealed marked recoil of the covered stent, resulting in significant residual stenosis. (**D**) Micro-computed tomography images of stent profile from the distal main and side branch orifices.

**Figure 3:**
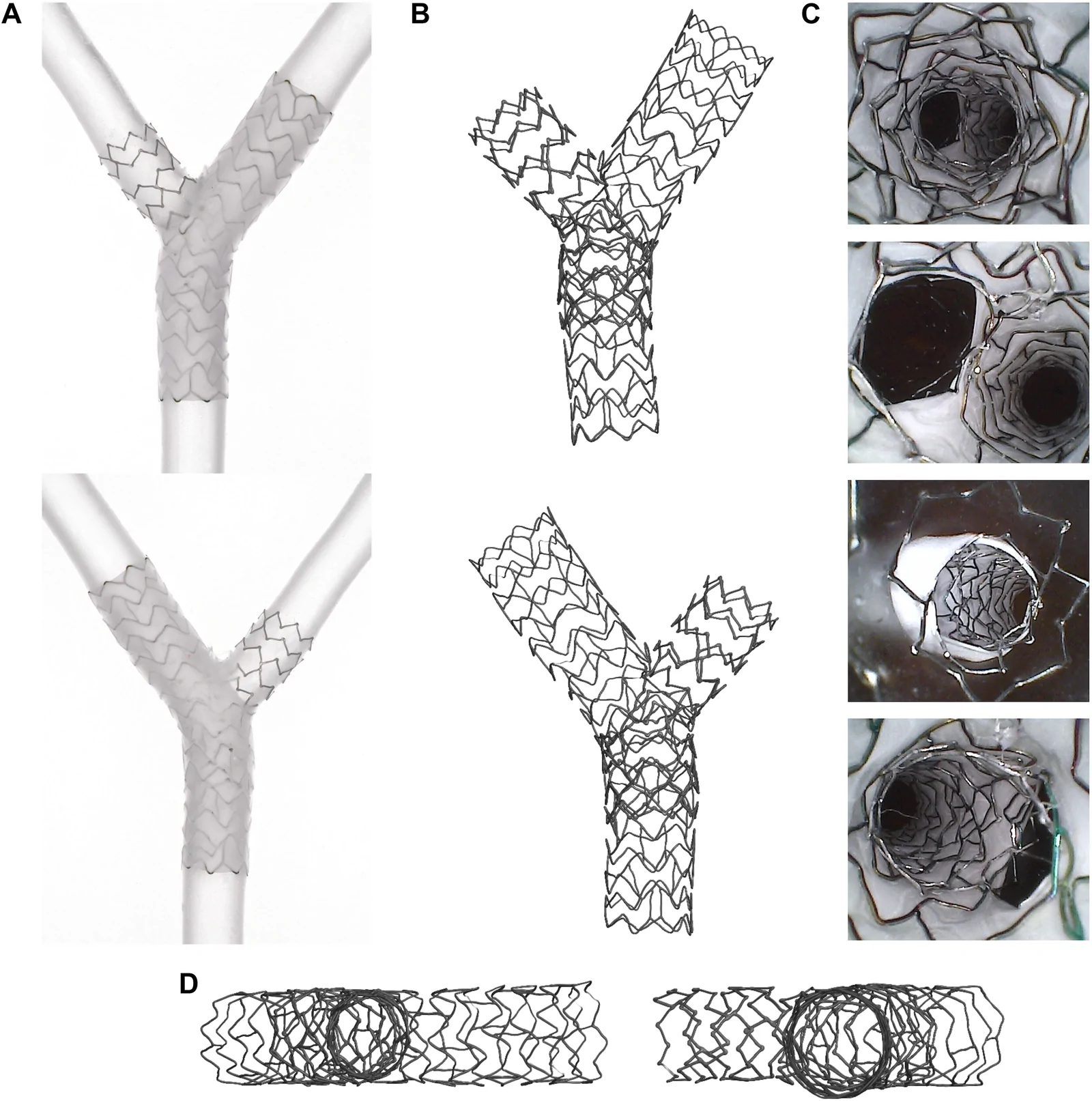
**Visual assessment of PK-Culotte.** (**A**) Macroscopic images of the covered stent. (**B**) Micro–computed tomography images of the covered stent. Combined visual inspection and micro–computed tomography imaging demonstrated generally acceptable structural integrity. (**C**) Endoscopic evaluation. The upper 2 images depict views from the proximal main branch; the third image shows a retrograde view from the side branch; and the lowermost image presents a retrograde view from the main branch. Endoscopy revealed that a second stent from the main vessel into the side branch can withhold the recoil to some degree. (**D**) Micro-computed tomography images of stent profile from the distal main and side branch orifices.

**Figure 4:**
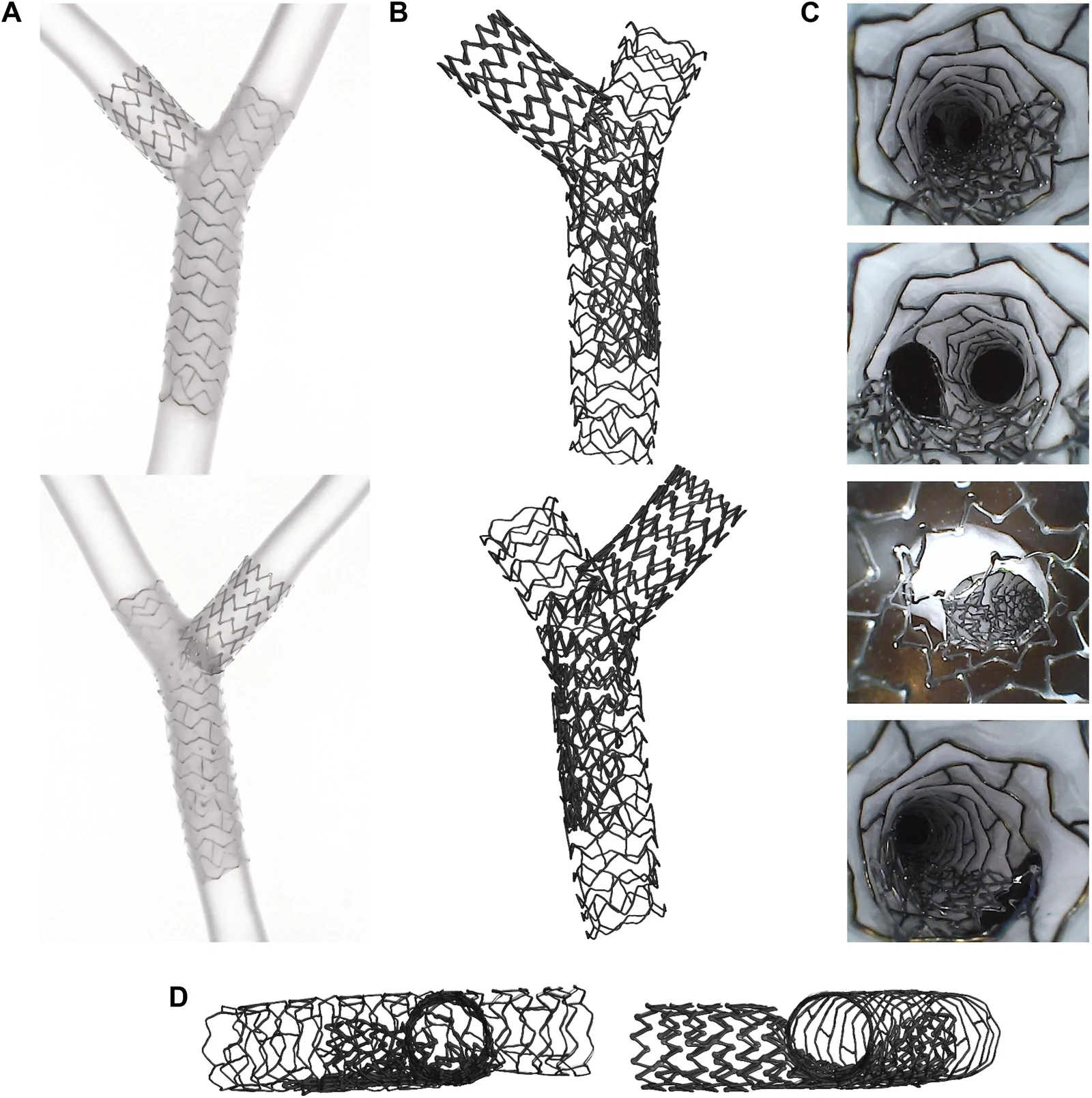
**Visual assessment of PK-Crush.** (**A**) Macroscopic images of the covered stent. (**B**) Micro–computed tomography images of the covered stent. Combined visual inspection and micro–computed tomography imaging demonstrated generally acceptable structural integrity. (**C**) Endoscopic evaluation. The upper 2 images depict views from the proximal main branch; the third image shows a retrograde view from the side branch; and the lowermost image presents a retrograde view from the main branch. Endoscopy revealed that a second stent from the main vessel into the side branch can withhold the recoil to some degree. (**D**) Micro-computed tomography images of stent profile from the distal main and side branch orifices.

**Figure 5:**
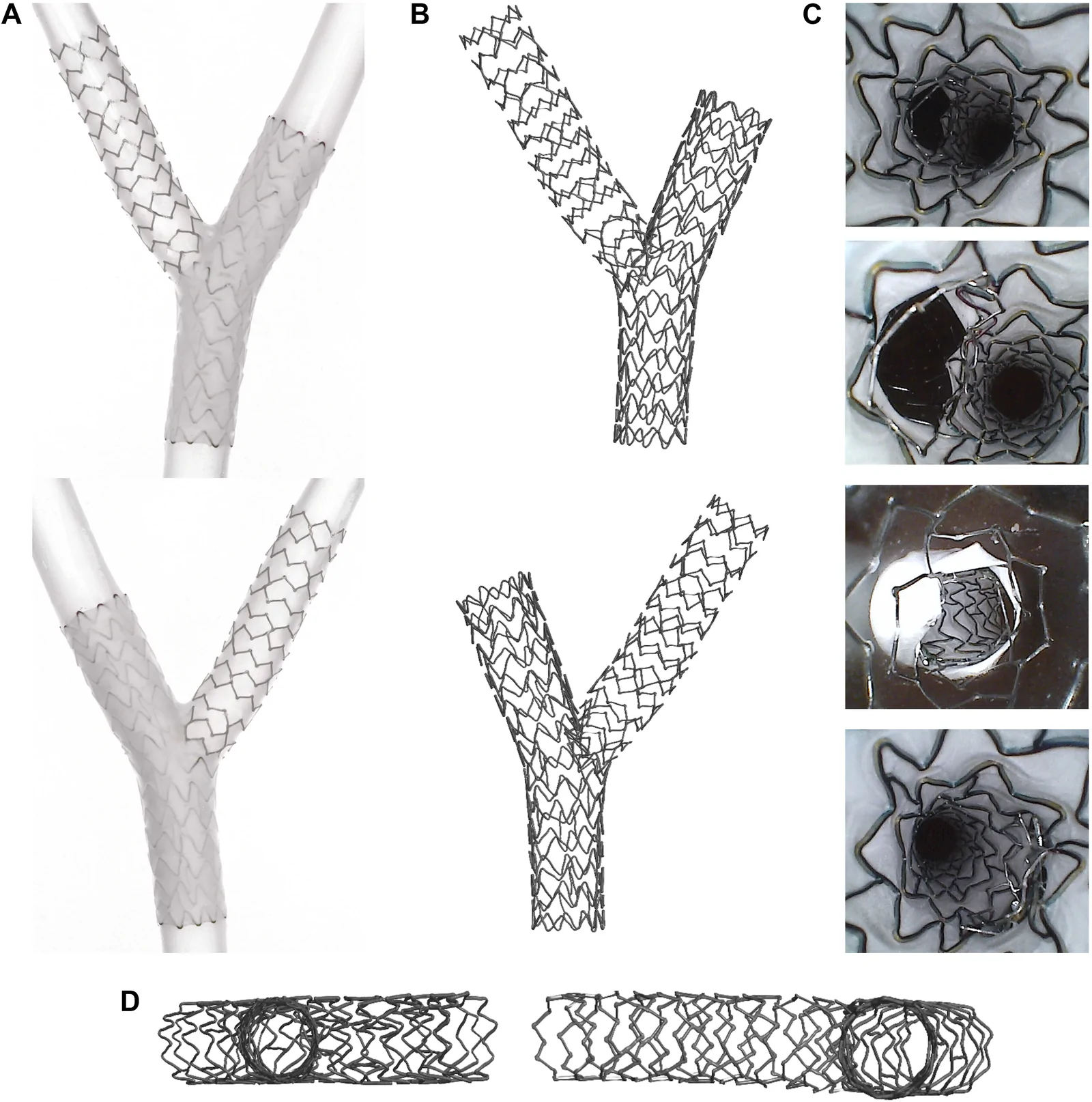
**Visual assessment of PK-Tap.** (**A**) Macroscopic images of the covered stent. (**B**) Micro–computed tomography images of the covered stent. Combined visual inspection and micro–computed tomography imaging demonstrated generally acceptable structural integrity. (**C**) Endoscopic evaluation. The upper 2 images depict views from the proximal main branch; the third image shows a retrograde view from the side branch; and the lowermost image presents a retrograde view from the main branch. Endoscopy revealed that a second stent from the main vessel into the side branch can withhold the recoil to some degree. (**D**) Micro-computed tomography images of stent profile from the distal main and side branch orifices.

**Figure 6:**
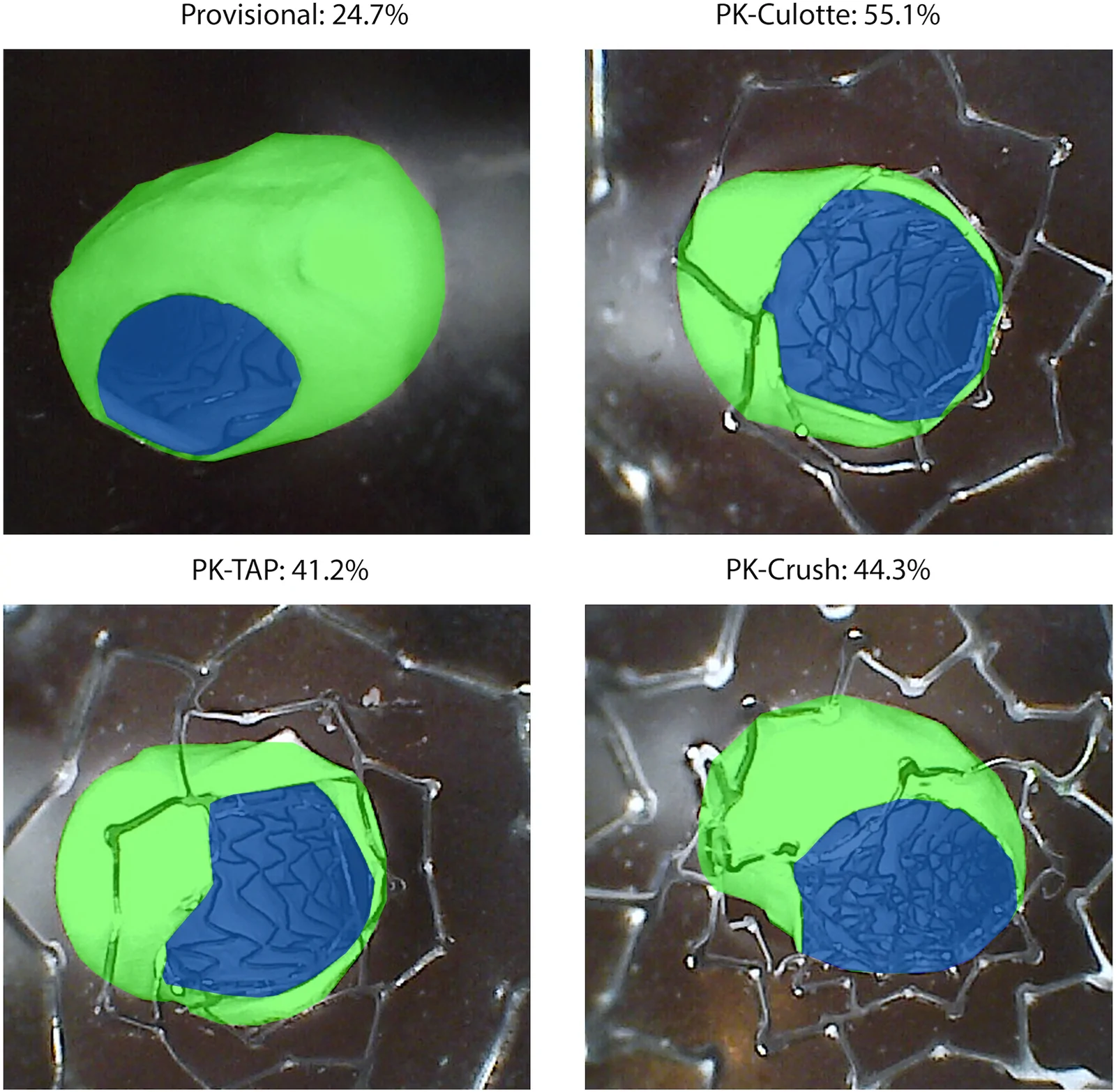
**Orifice segmentation.** Retrograde endoscopic imaging from the side branch. The polyurethane membrane is segmented in green, and the fenestrated (opened) area is highlighted in blue. The proportion of the opening was quantified by dividing the number of blue pixels by the total number of pixels in the membrane covering the side branch. The proportion of opened area is expressed as a percentage of the lumen area.

**Figure 7:**
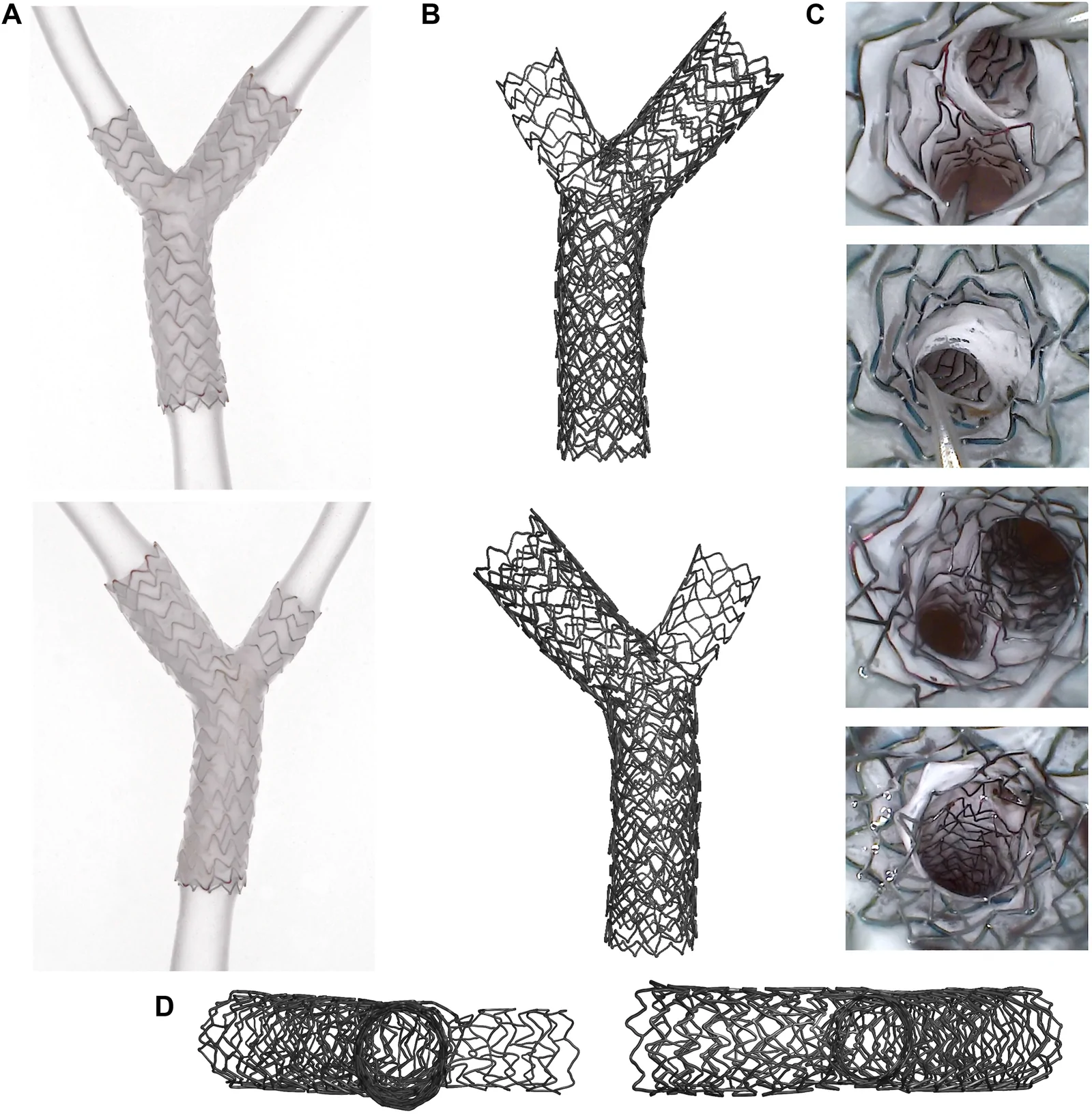
**Visual assessment of double PK-Culotte.** (**A**) Macroscopic images of the covered stent. (**B**) Micro–computed tomography images of the covered stent. Combined visual inspection and micro–computed tomography imaging demonstrated generally acceptable structural integrity. (**C**) Endoscopic evaluation. The upper 2 images depict views from the proximal main branch; the third image shows a retrograde view from the side branch; and the lowermost image presents a retrograde view from the main branch. Endoscopy revealed that a second stent from the main vessel into the side branch can withhold the recoil to some degree. (**D**) Micro-computed tomography images of stent profile from the distal main and side branch orifices.

### Clinical translation of bench-test findings

A 78-year-old man underwent elective PCI of the LM artery for recurrent angina 3 months post-NSTEMI (Figure 8A). Given his comorbidities, mantle cell lymphoma with grade III anemia and nursing home residence, PCI was performed via right radial access using a 7F sheathless guide. After implanting a Megatron 4.0 × 16 mm stent from LM to LAD (Figure 8B) and performing proximal optimization with a 6.0 mm NC balloon at 16 atm (Figure 8C), the patient developed chest pain and hemodynamic instability. Angiography revealed an Ellis type III perforation from the proximal LCx (Figure 8D). A drug-eluting stent was immediately deployed across the LCx perforation site; however, contrast injection showed persistent extravasation at the distal LM (Figure 8E). A Papyrus 4.5 × 20 mm covered stent was implanted from LM to LAD, successfully sealing the rupture but causing acute LCx occlusion with chest pain and electrocardiogram changes (Figure 8F). Initial attempts to fenestrate the covered stent with a Confianza wire failed because of unfavorable LM-LCx angulation. A SuperCross 120° microcatheter enabled successful wire crossing through the membrane. Sequential balloon dilations (1.0-2.5 mm) created a controlled orifice, allowing LCx stent deployment. After kissing balloon inflation and final re-POT with a 5.5 mm NC balloon, optimal angiographic results were achieved (Figure 8G). Total procedure time was 44 minutes; perforation-to-final result was 9 minutes. At the 6-month follow-up, the patient remained asymptomatic without recurrent events.

**Figure 8:**
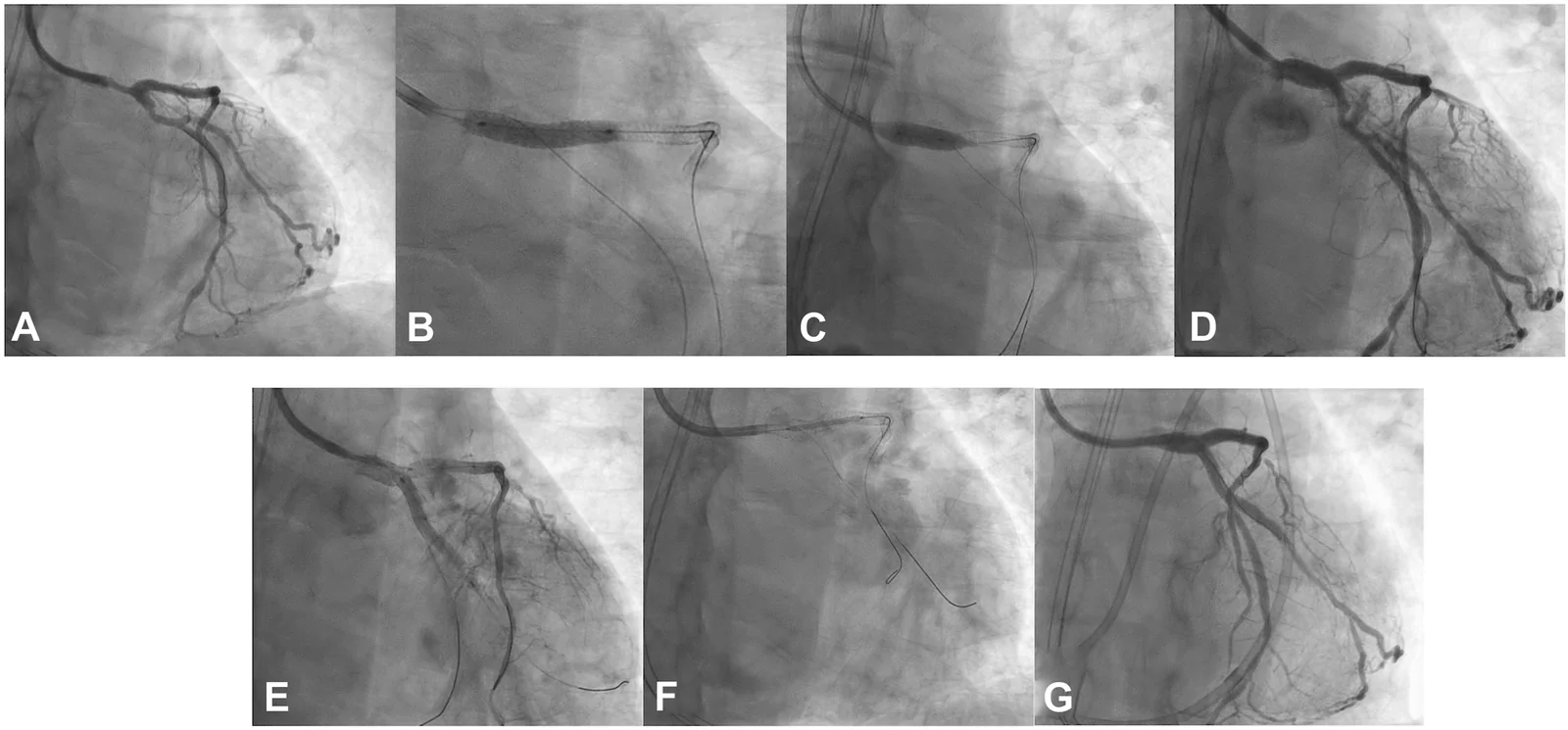
**Angiogram of the case report.** (**A**) Baseline angiography showing 80% left main (LM) stenosis with prior left anterior descending (LAD) stents visible at the ostium and a severe proximal left circumflex (LCx) stenosis. (**B**) Stent deployment from LM to LAD (Megatron 4.0 × 16 mm). (**C**) Noncompliant balloon inflation during POT. (**D**) Ellis type III perforation seemingly arising from proximal LCx with contrast extravasation. (**E**) Conversion to a TAP (T-and-protrusion) strategy, implanting an Xience 3.0 × 23 mm stent from LCx into the LM. Despite this, persistent contrast extravasation is visible at the distal LM level. (**F**) Implantation of Papyrus 4.5 × 20 mm covered stent sealing the perforation but occluding LCx. (**G**) Final angiography demonstrating patent LAD and LCx with no residual leak.

**Central Illustration:**
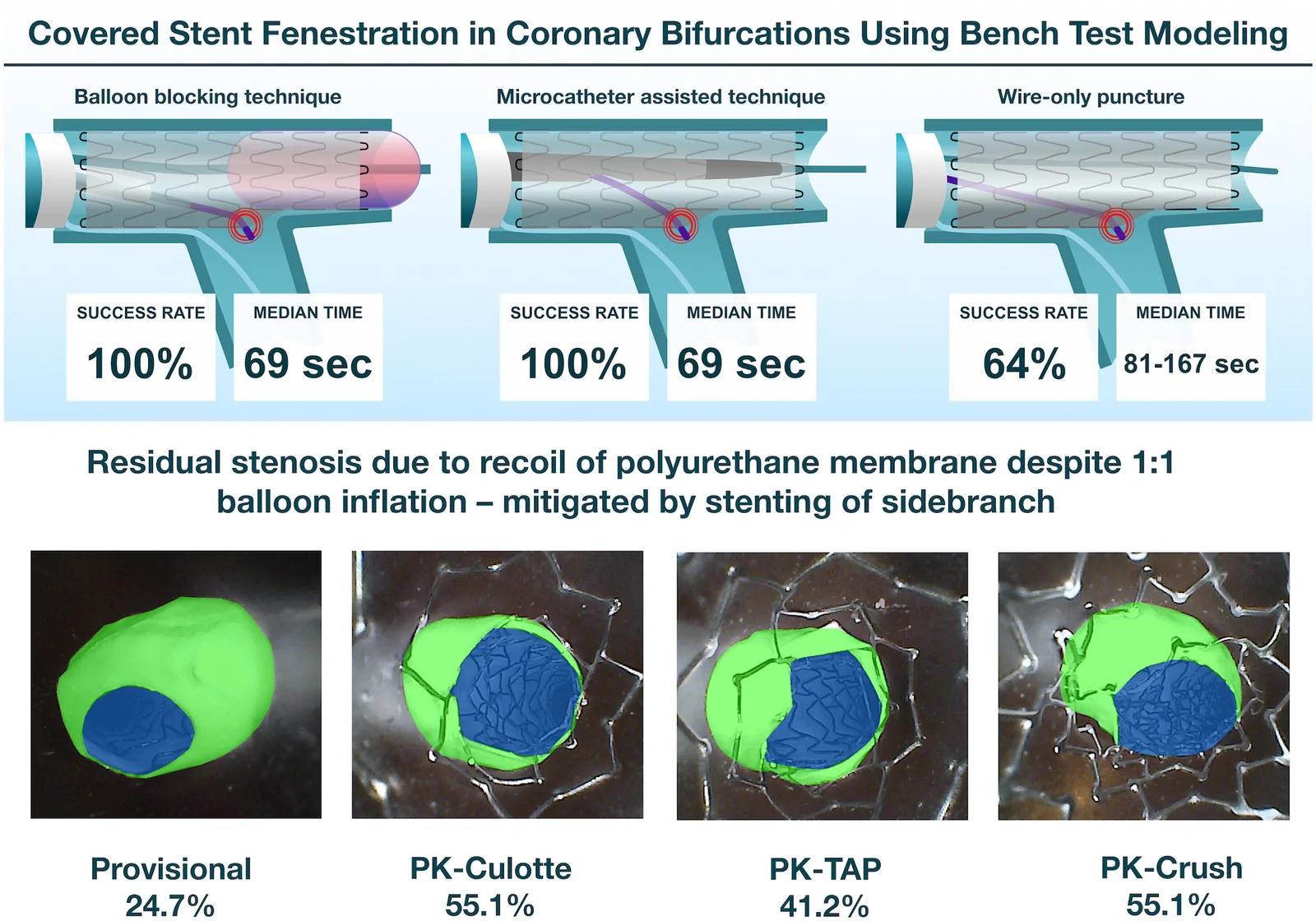
**Covered stent fenestration in coronary bifurcations using bench test modeling.** The upper panels illustrate quantitative metrics for the balloon-assisted blocking technique, the microcatheter-assisted technique, and the wire-only technique used for fenestration of covered stents. The lower panels present segmentation results derived from retrograde endoscopic imaging of the side branch. The polyurethane membrane is segmented in green, while the fenestrated (opened) area is highlighted in blue. The images demonstrate substantial residual stenosis, which is partially alleviated by a second procedural step, for which the Culotte technique is recommended.

## Discussion

This study provides a comprehensive bench-testing evaluation of covered stent implantation bailout technique for coronary perforations at the bifurcation level, while uniquely extending these findings to real-world clinical scenarios. Previous case reports have demonstrated the possibility to fenestrate covered stents with the use of double lumen or angled microcatheters and stiff wires.^6^, ^7^, ^8^, ^9^, ^10^, ^11^, ^12^, ^13^ In addition, a dual-covered stent “kissing” technique has been reported in one case to preserve SB perfusion,^14^ while another study described the use of a jailed-balloon approach as an alternative strategy.^15^ In one study, retrograde access through septal collaterals was employed as an alternative approach.^10^ However, the current literature lacks a detailed procedural methodology regarding the most effective steps, optimal material selection, and reproducibility of outcomes. We conducted the present study to address this gap. The outcomes of our experimental work can be summarized in 5 main conclusions. First, a cornerstone of this bailout technique is precise navigation, facilitated by anatomic or device-based landmarks to identify the course of the SB once it is covered by a covered stent. In all procedures, we had a guide wire in the SB that was jailed by the covered stent. This wire provided both visual guidance and optimized the trajectory for reentry. Such an up-front dual-wire approach is recommended in bifurcation and complex PCI. In the absence of a prepositioned SB wire, bailout strategies of this kind become substantially more challenging unless the SB has already been stented. Second, our preparatory work showed that soft-tipped guide wires with lower tip load were unable to fenestrate the polyurethane membrane and that a minimum tip load of 3 g was necessary. Third, fenestration was consistently achieved when applying a high-frequency “drilling/rotation” motion at the guide wire tip to penetrate the polyurethane membrane. Directional advancement techniques (“picking”) proved unsuccessful during the preparatory stages. Fourth, we show that multiple approaches, including wire-only, balloon occlusion, and dual-lumen microcatheter techniques, are feasible. Fifth, regardless of the initial technique, once fenestration is achieved and the patient has been hemodynamically stabilized, a definitive exit strategy is essential due to residual stenosis in the side branch (Central Illustration). This may involve completion of PCI with optimized stent reconstruction, in which a 2-stent technique will be required to counteract the inherent recoil of the polyurethane membrane, or, in operable patients, once they have stabilized, surgical revascularization may be an alternative.

### Choosing the right equipment

All previous studies reporting fenestration have used dual-lumen or angled microcatheters in combination with high-penetration guide wires, including the Halberd (Asahi Corp), Horton (Boston Scientific), Astato 20 (Asahi Corp), and Confianza Pro 12 (Asahi Corp).^6^, ^7^, ^8^, ^9^, ^10^, ^11^, ^12^, ^13^ We now show that it can be done with wires with as low a tip load as 3 g. However, other wire properties than just tip load may be of importance. In wire-only experiments, fenestration attempts with the ULTIMATEbros 3 were unsuccessful. However, fenestration attempts with the MIRACLEbros 3, having the same tip load, were successful. Although the ULTIMATEbros 3 tip is uncoated, the MIRACLEbros 3 features a hydrophobic, silicone-coated tip, which may facilitate engagement and penetration of the polyurethane material. The Judo 6 wire, although similarly hydrophilic to the ULTIMATEbros 3 and having a higher tip load, also differs by having a sharper tip, which may also influence fenestration performance. Ultimately, the choice of equipment should be guided by the operator’s expertise and procedural familiarity. However, when such experience is lacking, initiation with a 6 g wire appears to provide an appropriate balance between efficacy and safety.

### Choice of strategy

Our findings demonstrate that fenestration of a covered stent to restore SB patency can be achieved using multiple techniques. The balloon occlusion strategy is one such approach that can be systematically trained and reproducibly performed, offering an accessible option within the interventional armamentarium. Its key advantages include high reproducibility across operators with varying experience, reliance on widely available PCI equipment, low procedural cost, and ease of implementation because of clear fluoroscopic landmarks. However, a major limitation is the induction of transient ischemia in both the main and SB, particularly in unprotected LM interventions, where it can risk hemodynamic compromise. Ultimately, the choice of technique should be guided by the operator’s expertise and procedural familiarity. As a general principle, strategies should remain as straightforward as possible, while allowing for sufficient complexity when dictated by the clinical situation. The experimental design of this study did not permit direct statistical comparison between methods. Therefore, we cannot determine which technique is statistically superior. However, based on our experience when conducting these experiments, we recommend that operators use the technique they are most experienced with. For LM bifurcations, we suggest starting with a medium-stiff wire, either alone or supported by a microcatheter. For more distal bifurcations, the balloon occlusion strategy could be preferable.

### A 2-stent strategy is required to finish the job

A key finding of our study, which was presented for the first time at EuroPCR 2025, is the pronounced elastic recoil of the fenestrated segment, which persisted despite repeated balloon inflations, including cutting balloon inflations.^16^ This results in a reduction in the effective cross-sectional area of the SB ostium by up to 75%. In a clinical setting, this temporary restoration of flow may be sufficient to stabilize the patient and allow time for emergency coronary artery bypass grafting, but if bypass surgery is not feasible, definitive treatment with conventional stent implantation at the SB ostium becomes necessary. Based on our observations, the PK-culotte configuration appears to be the most effective technique for optimizing SB patency. However, the selection of a 2-stent strategy should be determined by the same anatomic and procedural considerations that inform conventional bifurcation stenting, including branch angulation, vessel size of both the main and SB, and overall lesion complexity. Some case reports have used ad hoc SB stenting, which was reported in a few studies, most commonly using TAP or culotte techniques.^7^^,^^11^^,^^13^ Maznyczka et al^6^ further highlighted the challenge of restenosis in an overstented circumflex artery, which required reopening with a stiff wire. In that case, OCT imaging revealed remnants of the polyurethane membrane protruding into the circumflex ostium, necessitating restenting with a reverse culotte technique. This was recently shown in bench testing by a different group.^17^ Holck et al^9^ reported a complex case of carinal perforation managed with a double Papyrus culotte technique. Insights from our experimental work suggest that in such scenarios, the use of an additional drug-eluting stent is required to counteract recoil following fenestration of the main vessel.

### Case report

The clinical case illustrates how prior bench-test simulations shaped real-time decision-making during an LM perforation. In this case, the operator was familiar with wire-membrane interaction, which enabled swift fenestration, while prior recognition of the polyurethane “curtain-drop” recoil phenomenon prompted early side-branch stenting, avoiding repeated inflations, excess contrast use, procedural delay, and operator uncertainty. We believe that bench tests can offer a reproducible platform for rehearsing complex bailout maneuvers, developing structured cognitive checklists, and internalizing clear escalation pathways. This study, therefore, advocates for simulation-based bench testing not merely as a research tool, but as a core educational strategy with demonstrable impact on real-world clinical decision-making.

### Limitations

Several limitations of this study warrant consideration. First, the experimental design does not permit direct statistical comparison between techniques, and no conclusions can, therefore, be drawn regarding the superiority of any single method. Second, the bench-test model, although high-fidelity, cannot fully replicate the complexity of real-world PCI, in which successful translation of these procedures to clinical practice requires advanced guide wire manipulation skills, typically at a level comparable to those used in chronic total occlusion interventions. Factors such as severe coronary calcification, extensive dissection, tortuous anatomy, carina shift, risk of wire perforation, and hemodynamic instability may substantially increase the technical difficulty of covered stent fenestration and SB rescue. Intentional membrane disruption may also increase the risk of incomplete sealing, particularly in calcified or irregular lesions, but this risk has to be weighed against the myocardium at risk of occluded SB. All of these factors fall outside the scope of the current study but are clinically relevant, as operators facing this complication must often choose between accepting a significant iatrogenic myocardial infarction or attempting a high-risk bailout strategy under adverse conditions.

## Conclusion

Covered stent fenestration is a feasible and reproducible bailout strategy for SB restoration following critical bifurcation perforations in a bench-test setting. However, the characteristic “curtain-drop” recoil of the polyurethane membrane at the fenestration site significantly reduces the effective ostial area despite adequate balloon inflation. Definitive SB patency, therefore, requires either emergency bypass surgery or additional conventional stent implantation at the SB ostium. This study demonstrates that knowledge and technical reflexes acquired through simulation-based bench testing can translate directly into real-world clinical practice, as evidenced by the successful management of a life-threatening LM perforation. These findings support high-fidelity bench testing as an essential educational strategy for preparing operators to confidently manage rare but catastrophic complications, bridging the gap between controlled simulation and critical real-world decision-making in contemporary interventional cardiology.

## CRediT authorship contribution statement

**Troels Yndigegn:** Writing – review & editing, Writing – original draft, Validation, Project administration, Methodology, Conceptualization. **Per Bondesson:** Writing – review & editing, Conceptualization. **Göran K. Olivecrona:** Writing – review & editing, Validation, Methodology, Conceptualization. **Henrik Wagner:** Writing – review & editing, Validation, Conceptualization. **Sasha Koul:** Writing – review & editing, Validation, Methodology, Conceptualization. **Matthias Götberg:** Writing – review & editing, Validation. **Helle Soeholm:** Writing – review & editing, Validation. **David Erlinge:** Writing – review & editing. **Mihai Cocoi:** Writing – review & editing, Visualization. **Olivier Gach:** Writing – review & editing. **Adrien Jossart:** Writing – review & editing, Validation. **Quentin Trefois:** Writing – review & editing, Validation. **Agostino Spano:** Writing – review & editing, Validation. **Giuseppe Colletti:** Writing – review & editing, Validation. **Gregor Leibundgut:** Writing – review & editing. **Marouane Boukhris:** Writing – review & editing. **Claudiu Ungureanu:** Writing – review & editing, Writing – original draft, Validation, Resources, Project administration, Methodology. **Moman A. Mohammad:** Writing – review & editing, Writing – original draft, Visualization, Validation, Software, Resources, Project administration, Methodology, Investigation, Formal analysis, Conceptualization, Writing – review & editing, Writing – original draft, Visualization, Validation, Resources, Project administration, Methodology, Investigation, Funding acquisition, Formal analysis, Conceptualization.
